# Repeated use of medications for ovulation induction after unsuccessful treatment: A cause of concern for infertile couples

**DOI:** 10.18502/ijrm.v18i1.6205

**Published:** 2020-01-27

##  Dear Editor,

The field of assisted reproductive technologies (ARTs) is rapidly progressing with many new advances to help infertile or sub-fertile couples to conceive and finally give birth (1, 2). The application of ARTs has solved the problems associated with infertility in many couples and has improved the results of infertility treatment. More than 5 million children have been born through these technologies and about 40 million couples have benefited after ARTs worldwide (3). However, ARTs may be associated with complications in women and fetuses after taking prescribed drugs (4). The side effects of different medications cause concern in couples who try several times to conceive with ARTs.

In a qualitative study, which was conducted on the experiences of 29 infertile couples with unsuccessful treatment using face-to-face in-depth interviews, many participants expressed their experiences of the immediate and long-term complications of medications used for ovulation induction. The immediate complications included hyper stimulation, frequent and painful injections, and the local complications of progesterone injections, which is discussed in more detail:

(1) Some participants experienced ovarian hyperstimulation after taking prescribed drugs. The ARTs may be associated with severe ovarian hyperstimulation syndrome (OHSS) (5). OHSS is a rare and potentially life-threatening problem that occurs in the process of infertility treatment (6).

(2) The majority of participants in the study suffered from frequent and painful injections. Infertility treatment is hard and frustrating, but infertile women tolerate it because of the value of its outcome (7). Our study showed that some infertile women were upset to restart painful procedures after unsuccessful treatment.

(3) Many participants complained of the local complications of progesterone injections that cause a stiff and painful injection site. One of the participants said that she had hives after the injection of drugs and needed to use corticosteroids and so was hospitalized.

The long-term effects of taking medications in our study included thromboembolic events, weight gain, and birth defects, which has been ellaborated below:

(1) Thromboembolic events may be among the long-term effects of taking medications. It is very important to identify the risk factors for thromboembolic events in OHSS patients. Changes in the hemostatic system increase the high concentration in women with severe OHSS (6).

(2) Weight gain caused by drug usage was another long-term effect of using ARTs, which was elaborated by some participants.

(3) Another long-term effect of ARTs emphasized by some participants was birth defects. One of the participants after two failures of the in-vitro fertilization (IVF) attempt, as well as because of observing the side effects of drugs on the fetus of one of their counterparts, gave up the treatment due to the fear of these side effects on her own fetus. Some infertile couples may be afraid of birth defects, as some studies suggest that birth defects in ARTs-babies are greater than those born with normal pregnancy (3, 8, 9). They were concerned about the lack of formation of cochlear in IVF-born babies. In a study, it was shown that ARTs can lead to hearing impairment and ear disorders, such as cases of bilateral lumbar puncture in the ear, one-sided lumbar puncture from the tympanum, and inflammation of the unilateral and bilateral tympanic membrane (10). ARTs increase the risk of Beckwith-Wiedemam syndrome by 10% and can also be involved in the pathogenesis of genomic diseases in addition to methylation abnormalities (11). Factors such as the lack of choice of natural mechanisms in ARTs-induced pregnancies, hormonal changes in the laboratory as the cause of aneuploidy chromosomes, and spot mutations due to exposure to chemicals in the laboratory may increase congenital abnormalities in ARTs-babies (9). Also, in these methods, many drugs which are prescribed for ovulation induction, egg retrieval and embryo culture, freezing and melting embryo under laboratory conditions, and using high doses of progesterone to support luteal phases, all may cause damages to gametes or embryos (3). Therefore, it seems that the infertile couples' fear of the possibility of developing defects in embryos caused by ARTs is a logical challenge for those who undergo multiple treatments using ARTs. A meta-analysis also showed that ART methods are introduced as a risk factor for placenta previa (12) and preeclampsia (13).

We argue that these findings have presented the experiences of Iranian mothers in the context of Iranian infertility clinics, however, it seems that the experiences of our infertile couples could be similar to others across the world, regarding their experiences with medications in IVF process. To sum up, although the studies have shown the maternal and fetal complications with IVF (3, 12), no attention is drawn to infertile women's concern on this topic. It seems that it will be useful to develop clinical practice protocols and guidelines to improve OHSS, reduce multiple pregnancies, and increase live birth rates (1). Counseling and supportive interventions such as collaborative infertility counselling model, cognitive-behavioral intervention, and psychosocial interventions may decrease the mentioned concerns and stress in ARTs (14, 15) and as a consequence could help infertile women to cope better with the infertility stress (16, 17). Also, it is necessary to do further research in relation to the complications of ARTs.

**Samira Ebrahimzadeh Zagami^1comma2^ Ph.D., Roksana Janghorban^3^ Ph.D., Robab Latifnejad Roudsari^1comma2^ Ph.D.**

^1^Nursing and Midwifery Care Research Center, Mashhad University of Medical Sciences, Mashhad, Iran.

^2^Department of Midwifery, School of Nursing and Midwifery, Mashhad University of Medical Sciences, Mashhad, Iran.

^3^Maternal-Fetal Medicine Research Center, Shiraz University of Medical Sciences, Shiraz, Iran.

##  Conflict of Interest

None.
